# Recent advances in influenza vaccines

**DOI:** 10.12688/f1000research.22611.1

**Published:** 2020-04-28

**Authors:** Graham Pawelec, Janet McElhaney

**Affiliations:** 1 Department of Immunology, University of Tübingen, Tübingen, Germany; 2 Health Sciences North Research Institute, Ontario, Canada

**Keywords:** influenza, vaccine, adjuvant, effectiveness, elderly

## Abstract

Seasonal influenza remains a major public health problem, responsible for hundreds of thousands of deaths every year, mostly of elderly people. Despite the wide availability of vaccines, there are multiple problems decreasing the effectiveness of vaccination programs. These include viral variability and hence the requirement to match strains by estimating which will become prevalent each season, problems associated with vaccine and adjuvant production, and the route of administration as well as the perceived lower vaccine efficiency in older adults. Clinical protection is still suboptimal for all of these reasons, and vaccine uptake remains too low in most countries. Efforts to improve the effectiveness of influenza vaccines include developing universal vaccines independent of the circulating strains in any particular season and stimulating cellular as well as humoral responses, especially in the elderly. This commentary assesses progress over the last 3 years towards achieving these aims. Since the beginning of 2020, an unprecedented international academic and industrial effort to develop effective vaccines against the new coronavirus SARS-CoV-2 has diverted attention away from influenza, but many of the lessons learned for the one will synergize with the other to mutual advantage. And, unlike the SARS-1 epidemic and, we hope, the SARS-CoV-2 pandemic, influenza will not be eliminated and thus efforts to improve influenza vaccines will remain of crucial importance.

## Introduction

Despite the common perception that it is “only a flu”, seasonal influenza is a powerful pathogen responsible for many hundreds of thousands of deaths every year, especially of elderly people. It is somewhat puzzling that a highly contagious pathogen responsible for an estimated average of half a million fatalities every year is faced with such insouciance by most people. Indeed, globally, 300,000 to 700,000 people die from the consequences of respiratory complications of influenza each year, with a huge difference in mortality rates according to age: possibly a vanishingly small number of younger adults (0.001%) compared with 0.03% of people aged 65–74 but rising to 0.1% of people over 75 ^1^. Although this may seem to be a low chance of death caused directly by influenza, indirect sequelae of influenza infection contribute to a deteriorated health status and frailty in the elderly. These long-term sequelae are not limited to the respiratory tract but are increasingly associated with systemic, especially cardiovascular ^2^, symptoms.

Vaccination is a highly effective, minimally invasive, and cheap protective measure. However, there are many unsolved problems associated with the current seasonal influenza vaccines. Thus, advances in effective influenza vaccination must include increasing the protective efficiency of the vaccine, especially in the elderly. This review will focus on recent advances in the science and the R&D, but one should not forget that the sociology of enhancing acceptance and uptake is of paramount importance too.

## Disadvantages of current influenza vaccines and efforts to improve them

Predicting the next season’s predominant influenza strains is a major undertaking that is always fraught with difficulty and often incorrect. Recent advances in surveillance, data exchange, and bioinformatics may help to mitigate this problem. Unexpectedly, help may be at hand from the surveillance of social networking sites, which may yield more topical data than public health services ^3^. However, instead of chasing seasonal variations, it would be much more advantageous to develop vaccines that were effective against all influenza strains, hence the intensive efforts to develop “universal vaccines” that will protect regardless of the seasonal strain. Many such efforts focus on directing antibody production away from targeting parts of the virus that are different from strain to strain (i.e. the highly variable hemagglutinin [HA] head structures) and towards generating antibodies against conserved antigens. These may be from the stem part of the molecule, which is not normally immunodominant. These antibodies should provide heterosubtypic protection, i.e. against multiple different strains ^4^. However, until recently, this approach was limited to one viral group, but modifying the glycosylation state of stem regions may increase antibody accessibility and broaden the range of strains targeted ^5^. It is not known whether these experiments in mice or even in ferrets reflect what would be effective in humans. In naturally acquired H1N1 infection, at least, it seems that anti-head and not anti-stalk antibodies play a predominant role, as expected ^6^. Nonetheless, in passive immunisation studies, monoclonal antibodies against stem antigens can be protective in human influenza challenge ^7^. Other approaches include active immunisation with multi-epitope protein vaccines containing several from influenza A and B, common to multiple strains of influenza virus. There is some evidence in humans that vaccination in one season may confer protection in the next season against strains that were not circulating at the time of the earlier vaccination. An analogous approach employing mixtures of synthetic peptides is also being pursued by other companies, for example ^8^. An advantage of this approach, analogous to that in cancer vaccines, is to select epitopes stimulating both humoral and cellular immunity; indeed, an interesting aspect of the action of the M-001 vaccine ^9^ is that it does not contain any HA head epitopes and stimulates predominantly cellular responses. Another approach attempts to exploit an elegant idea to focus antibody responses on the stalk by vaccinating with stalk domains engineered onto different head domains, which were shown to be protective in mice ^10^. A very recent publication now reports the outcome of a phase I clinical trial concluding that high anti-stalk titres were induced and paves the way for further development of universal influenza virus vaccines ^11^.

## Rapidity and volume of vaccine production

A major bottleneck in influenza vaccine production is the inability to generate large amounts of vaccine quickly. One problem here resides in methodology for producing the vaccine, which requires improvement. The technique still employed by most vaccine producers is to grow the virus in hens’ eggs. Alternatives are being energetically sought after, including cell culture approaches and genetic engineering of viral components. There are several reasons for this, not only to speed up the cumbersome process of growing the virus in eggs (not to mention allergy problems) but also because in some cases egg-adapted viruses used to make the vaccine are not identical to the wild-type pathogen in circulation that season and do not protect ^12^. Thus, it was concluded that the quadrivalent vaccine “Flucelvax” (grown in cultured canine kidney cells) was potentially superior to egg-based vaccines in real-life practise as well as in clinical trials ^13^, and importantly a similar trivalent vaccine was reported to be effective in individuals over 60 years of age ^14^. An alternative approach dispensing with the need for using live viruses has now reached fruition in the “FluBlok” vaccine using cultured insect cells to produce the vaccine after their infection with genetically engineered baculovirus vectors. Employing a direct comparison with egg-grown virus vaccines, researchers reported that the recombinant vaccine was more efficacious, also in older adults ^15^. The use of recombinant technology obviates the need for using live viruses, and once the sequence is known, production can be much more rapid, which is important for combating new and emerging strains. It may also be possible to simplify production even more by producing recombinant vaccines in plants, which can result in very rapid synthesis of viral proteins. Plant-based vaccines have not yet entered clinical trials, but experiments in mice have suggested that such virus-like particles are protective, even in very old animals ^16^. Some investigators go even further and propose that mRNA itself can be used as a vaccine without the need to produce viral proteins outside of the host at all. This results in probably the most rapid pipeline for developing prophylactic vaccines. To stabilise the RNA, it can be enclosed in liposomes, as in the mRNA-1851 phase I trials in Miami and Berlin, showing immunogenicity and good tolerance ^17^.

## Adjuvanted vaccines

Adjuvants are non-immunogenic vaccine components that enhance immunity in different ways: by a depot effect or by stimulating antigen-presenting cells, for example ^18^. Optimal vaccine formulations will of course depend on the route of administration. All of these considerations have been receiving a great deal of attention over the last 3 years. New data on the use of well-established adjuvants such as M59 or even alum reflect the rather surprising relative paucity of information on their action ^19, 20^. Work on developing new adjuvants, such as “self-adjuvanting” lipid nanoparticles including Toll-like receptor (TLR) ligands, is beginning to deliver encouraging results ^21^. Mouse models are revealing that responses to intranasal vaccines may also be enhanced by the inclusion of adjuvants such as the TLR ligand CpG ^22^. Increasing attention is being paid to testing new adjuvants not only in young animals but also in those of advanced age, the most susceptible group ^23^. Mouse models are suitable for testing the use of live attenuated viral vaccines, rather than the non-viable immunogens usually employed. Thus, the effects of different interferons on outcomes can be assessed using genetically deficient mice, for example ^24^. However, mice are not people, and proof of the pudding must be RCTs in humans, but there remain relatively few adjuvants in common use so far and this differs in different countries ^25– 27^. Developing better adjuvants is a high priority, especially for vaccination of the elderly ^28^. Current discussions centre around the practicalities and ethics of human challenge models for assessing vaccine efficacy in the most relevant possible “model” ^29^.

## Route of administration

Flu vaccines are mostly injected intramuscularly (i.m.), which may be suboptimal. Alternative routes include intradermal (i.d.), oral, or inhaled. Regarding the route of administration, oral vaccines using live attenuated viruses have not yet found widespread application since pilot studies in humans in 2016 ^30^, but in the meantime different approaches are being tested to protect various forms of immunogen from degradation in the stomach ^31, 32^. The expectation that i.d. administration might be more effective than the usual i.m. injection seems not to have been fulfilled, judging from the data thus far accumulated. For example, a comparison of recombinant vaccine followed by trivalent inactivated vaccine given i.d. or i.m. revealed no differences ^33^. There were some earlier data suggesting that for the elderly the i.d. route might be more efficacious, but differences were not large ^34^. Other variant application routes are being examined in mice, for example, the so-called “prime-pull” strategy whereby i.m. priming is boosted by inhalation ^35^. A very recent study employed intranasal administration of a novel adjuvanted vaccine to mimic natural influenza infection and to activate CD8 ^+^ T cells *in situ* in the lungs. This sophisticated approach employed 2’,3’-cyclic guanosine monophosphate–adenosine monophosphate (cGAMP) as an “adjuvant” to activate the innate immune sensor stimulator of interferon genes (STING) targeted to lung-resident alveolar macrophages and alveolar epithelial cells (AECs). Moreover, encapsulation of cGAMP with pulmonary surfactants enabled stimulation of STING in AECs while sustaining the barrier of the pulmonary surfactant appropriately. In this way, intranasally administered vaccine resulted in protection against multiple strains of influenza in mice and ferrets, associated with both humoral and cellular responses ^36^.

## Factors influencing effectiveness of vaccination

Current vaccines are licensed based on World Health Organization-approved centres that test efficacy solely in terms of standard measures of humoral immunity. Subjects do not necessarily include elderly people, who may not respond as well as younger adults, and differences in responses between older men and women ^37^ are not taken into account. Measured parameters defining responses on the basis of which vaccines are licensed may not always be appropriate: for example, the requirement for an increase in antibody titre to two of three strains in the vaccine may miss the third one that could be critical that season. Also, non-response could erroneously be attributed to subjects who already have a high (protective) titre of antibody pre-vaccination and cannot increase it more. These measures of vaccine responsiveness thus cannot predict clinical protection. In this context, advances in molecular analysis of the immune response may lead to insights on individual variability and guide vaccine design and application ^38, 39^. Hence, better predictive biomarkers are required, particularly considering the essential component of T cell immunity, which is not usually measured in the assessment of vaccine efficacy prior to licensing. Over the last 3 years, the realisation that vaccines need to be formulated to take the importance of the T cell response into account has come to the fore. This is a crucial issue not only because T cells are required to eliminate infected host cells prior to viral release but also because the epitopes recognised by T cells tend to be conserved across viral strains ^40^. Thus, efforts to develop new and improved vaccines increasingly aim to generate cellular as well as humoral responses ^41^. Moreover, the type of cellular response achieved is likely to be of major importance ^42^, and this can be markedly influenced by the nature of the adjuvant ^43^ as well as by multiple host factors including frailty ^28^ and medication ^44, 45^. The impact of frailty is currently being intensively investigated, with some studies clearly documenting an important influence on effectiveness ^46^ while others do not ^47^. The reasons for such discrepancies are likely to be multifactorial and are not yet clarified but may at least partly reside in definitions of frailty and pre-frailty ^48^ as well as the population studied and subject selection ^49^. In particular, there has been some controversy regarding whether repeated annual vaccinations with the same antigens might result in decreased responsiveness ^50^, although recent studies suggest that this is not likely to be the case ^51^. Confounding factors could also include exposures over the life-course that may have thus far under-investigated effects, such as exposure to ionising radiation ^52^. It has also been noted that obesity can dampen influenza vaccine efficiency (although, importantly, it did not decrease efficacy, i.e. the serological response) ^53^. Other important host factors probably include the influence of infection with HIV, even when controlled ^54^, or with other persistent latent viruses. One of the latter is most notably cytomegalovirus (CMV), which is also likely to play a role, although its impact on humoral responses remains controversial ^55^. This may at least partly be due to the fact that CMV infection mostly affects T cell responses, with only knock-on effects on antibody levels, especially in the elderly ^56^. The main mechanism responsible may be CMV-driven impaired granzyme B responses in influenza-specific cytotoxic CD8 ^+^ T cells and higher levels of IL10 ^57^, either human or CMV decoy derived ^58^. Finally, an impact of the microbiota, usually taken to refer to the gut microbiota, is emerging as an important confounding factor in responsiveness to influenza vaccination ^59^. Attempts to manipulate the microbiota to enhance responsiveness are being tested in clinical trials using probiotics ^60^ or synbiotics ^61^, so far without resounding success but with some recent evidence of efficacy in the elderly ^62^. An extensive “super-meta-analysis” of 28 studies published at the end of 2019 investigated the state of knowledge on the impact of “intravenous drug use, psychological stress, acute and chronic physical exercise, genetic polymorphisms, use of pre-/pro-/symbiotics, previous Bacillus Calmette-Guérin vaccination, diabetes mellitus, vitamin D supplementation/deficiency, latent CMV infection and various forms of immunosuppression” on responses to influenza vaccination ^63^. This study concluded that “while the inhibiting effect of several immunosuppressive host factors was evident, the enhancing effect of pro/pre/symbiotics and chronic physical exercise was doubtful and virus type-specific (A but not B)” and that “studying the host-related correlates of the influenza vaccine-induced immune response could contribute to the production of new personalized vaccines and to the development of new patient-oriented vaccination strategies in a value-based public health perspective” ^63^. Nonetheless, there are also detailed studies documenting improved cellular and humoral responses in elite athletes, so the degree of exercise and overall fitness may be crucial to seeing increased responsiveness ^64^. Thus, these issues all remain a focus of intensive research which undoubtedly carry a great deal of relevance not only for influenza vaccination but for responses to other pathogens as well. Moreover, benefits of effective vaccination to prevent influenza may be felt in unrelated areas of healthcare owing to their indirect impact on other diseases as diverse as lung cancer ^65^ and in particular cardiovascular disease ^66^.

## Cost of developing a new influenza vaccine and vaccine cost-effectiveness

While academic research teams are working on the development of “universal influenza vaccines” and other improvements and advances, one should not forget that the regulatory pathway to the approval of these new vaccines is often referred to as the “valley of death”: many new vaccines never make it through the many steps in the approval process. The cost of making a new influenza vaccine is estimated at $1 billion and takes 10–15 years. Even the cost of annually refreshing the influenza strains contained in the current seasonal vaccines is estimated to be $5–18 million per year. These are significant non-scientific hurdles that need to be overcome when considering recent advances in influenza vaccines ( https://www.wired.com/story/flu-vaccine-big-pharma/). Nonetheless, a recent analysis suggests that despite the drawbacks of current seasonal influenza vaccines, there is a huge public health and public financial benefit to the use of influenza vaccines so that further improvements would make a big impact ^67^. As vaccines produced in cells rather than eggs become more generally available, some analyses are concluding that in addition to other potential advantages (see above), they may also be more cost-effective ^68^.

## Perspectives: future scenario of personalised vaccination

As with other areas of medicine, a “one-size-fits-all” approach to influenza vaccination will never be optimal for every individual. In particular, the state of health and pre-exposures of the vaccinee will be highly influential in determining the success of the vaccine. In an ideal situation, prior to vaccination, the immunological history of the person would be assessed from a small blood sample. This would determine the state of humoral and cellular immunity as it pertained to influenza reactivity and the composition and nature of the vaccine modified accordingly. If the individual manifested problems regarding deficits in the presence and functions of antigen-presenting cells, steps would need to be taken to adjust adjuvants or vaccine antigens, or even replace defective antigen-presenting cells with artificial engineered constructs ^69^, which might one day be possible *in vivo*. Similarly, if the T cell or B cell repertoire lacked cells with the appropriate antigen receptors, these could be engineered in. This scenario is admittedly highly unlikely, even in the not-so-near future, but technological progress in these areas, partly driven by efforts of cancer therapy researchers ^70^, has been so rapid that such an individualised approach may become feasible at some point.

## Conclusions

In the past 3 years, there has seen steady progress in the science behind the development of improved influenza vaccines, but the main hurdles have not yet been overcome. These remain the continued necessity for producing seasonal vaccines rather than a universal vaccine, the mode of production (egg, cultured cells, recombinant products), the development of better adjuvants, the focus on humoral and under-appreciation of cellular immunity, and perceived problems of immunosenescence ^28, 71, 72^, as well as poor vaccine uptake in most countries. Although practical progress has been slow, results from the last 3 years encourage the belief that significant inroads will be made over the next 3 years.
